# Avian Host-Selection by *Culex pipiens* in Experimental Trials

**DOI:** 10.1371/journal.pone.0007861

**Published:** 2009-11-17

**Authors:** Jennifer E. Simpson, Corrine M. Folsom-O'Keefe, James E. Childs, Leah E. Simons, Theodore G. Andreadis, Maria A. Diuk-Wasser

**Affiliations:** 1 Department of Epidemiology and Public Health, Yale School of Public Health, New Haven, Connecticut, United States of America; 2 Department of Biology, Stony Brook University, Stony Brook, New York, United States of America; 3 The Connecticut Agricultural Experiment Station, New Haven, Connecticut, United States of America; London School of Hygiene & Tropical Medicine, United Kingdom

## Abstract

Evidence from field studies suggests that *Culex pipiens*, the primary mosquito vector of West Nile virus (WNV) in the northeastern and north central United States, feeds preferentially on American robins (*Turdus migratorius*). To determine the contribution of innate preferences to observed preference patterns in the field, we conducted host preference trials with a known number of adult female *C. pipiens* in outdoor cages comparing the relative attractiveness of American robins with two common sympatric bird species, European starling, *Sternus vulgaris* and house sparrow, *Passer domesticus*. Host seeking *C. pipiens* were three times more likely to enter robin-baited traps when with the alternate host was a European starling (n = 4 trials; OR = 3.06; CI [1.42–6.46]) and almost twice more likely when the alternative was a house sparrow (n = 8 trials; OR = 1.80; CI = [1.22–2.90]). There was no difference in the probability of trap entry when two robins were offered (n = 8 trials). Logistic regression analysis determined that the age, sex and weight of the birds, the date of the trial, starting-time, temperature, humidity, wind-speed and age of the mosquitoes had no effect on the probability of a choosing a robin over an alternate bird. Findings indicate that preferential feeding by *C. pipiens* mosquitoes on certain avian hosts is likely to be inherent, and we discuss the implications innate host preferences may have on enzootic WNV transmission.

## Introduction

Heterogeneities in contact rates between arthropod vectors and hosts are important to vector-borne disease dynamics, because they can result in increased disease transmission if vector blood meals occur more commonly on pathogen-competent hosts. In contrast, transmission may be reduced if blood meals are ‘diluted’ by feeding on non-competent hosts.

The rate of contact is influenced by a variety of factors including innate host preferences of the vector, host availability and landscape composition and configuration. Traditional means for determining mosquito host-preferences in the field are to estimate the proportion of blood meals on certain hosts [1] or, more specifically, to calculate a feeding index, where the proportion of blood meals acquired from a specific host is assessed in relation to the abundance of that host within the community of potential hosts [2], [3].

To control for environmental confounders influencing contact rates, host-choice experiments using traps baited with whole-host odors have been conducted to determine innate host preferences of a vector. Mainly focused on *Anopheles* spp. malaria vectors, these experiments have demonstrated preferential feeding for individuals of a certain host species [4]–[12]. A limited number of host-preference experiments conducted with other mosquito genera provide inconclusive evidence on the presence of innate host preferences [5], [8], [13]–[19].

In the United States, West Nile virus (WNV; family Flaviviridae, genus *Flavivirus*) is maintained in a zoonotic cycle involving mosquito vectors and wild birds serving as amplification hosts. Mammals, including humans, are generally considered non-competent or ‘dilution’ hosts because they do not develop viremias of sufficient magnitude and duration to infect mosquitoes and thus do not contribute to the transmission cycle [20]. WNV was first recognized in North America in 1999, and has since spread throughout the United States and southern Canada, causing more than 28,900 human cases and 1,131 fatalities (Centers for Disease Control and Prevention, 2008). In the northeastern US, the mosquito *Culex pipiens* L. (Diptera: Culicidae) is considered an important enzootic vector for WNV, as numerous isolations of WNV have been obtained from field-collected mosquitoes [21]–[24] and laboratory studies have confirmed its vector competence [25]–[27].

Recent studies in the northeastern US and elsewhere, have found that a single host species, the American robin (*Turdus migratorius*), provides between 5 and 71% of all *C. pipiens* blood meals, with most reporting higher than 40% robin-derived blood meals [28]–[35]. Kilpatrick et al [30] and Hamer et al [35] have further shown that robins are fed upon by *C. pipiens* in excess of what would be expected based on their relative abundance in the host community, as determined by a feeding index >1. Field derived feeding indices, also referred to as a ‘selection’ [35] or ‘forage’ [36] ratio, are, however, limited in their ability to discriminate between an ‘innate’ feeding preference and the effect of landscape composition and configuration on the contact rate between *C. pipiens* and robins.

The objective of this study was to obtain a measure of innate host-selection in *C. pipiens* by comparing the relative attractiveness of American robins when paired with two other bird species common in residential areas, European starling (*Sternus vulgaris*) and house sparrow (*Passer domesticus*), in outdoor caged experiments.

## Materials and Methods

### Mosquitoes

All *C. pipiens* mosquitoes used in the experimental host selection trials were reared from field collected egg rafts obtained in New Haven, CT between August 15 and September 29, 2008 using gravid-trap bins baited with a lactalbumin and yeast hay infusion. Egg rafts were collected daily and placed individually in 100×15mm Petri dishes filled with approximately 40ml purified water and a small amount of crushed flake fish food, at 70% relative humidity. Larval rearing was conducted at the Connecticut Agricultural Experiment Station's insectary located at Lockwood Farm in Hamden, CT at 22°C under a 16 hr photoperiod. *C. pipiens* larvae were readily identified to species during the second stadium using standard morphological characters with the aid of a stereo microscope and descriptive keys [37]. Three to 5 egg batches were then transferred simultaneously to larger 30×19cm pans filled with 100ml water for rearing to adulthood. Emerging adults were maintained in 30.5×30.5×30.5cm screened cages under the same relative humidity conditions and were provided with a 10% sucrose solution. Adult female *C. pipiens* used in the host selection trials were at least 4 days post-emergence and were deprived from the sucrose solution for 24 hr prior to each trial.

### Birds

All animal work was conducted in accordance with relevant national and international guidelines by the US Geological Survey (USGS) and the Ornithological Council. Necessary university (IACUC 2006-07596), state (0109017) and federal (MB122969-2) permits were obtained for this study to ensure appropriate care and handling of birds. All birds were captured in New Haven County using either mist-nets or starling traps (New Haven Troyer V-Top Repeating Sparrow and Starling Trap®, #6057) and were subsequently housed at the Yale Farm in Bethany, CT. Captured birds were identified to species, given a unique identifier (robins were banded) and classified by sex and age if possible. Birds were used in only one trial, held for no more than 24 hours, and all birds were successfully released the morning after the trial at the location of capture.

Prior to trials, captured birds were held individually in 83.3 or 143.8 liter mesh reptarium cages supplied with paper liners in a designated animal room at the Yale Farm at 24°C and 76.0% RH. Perches made of sticks with faux foliage were provided and birds were given unlimited water and ample food. Robins were provided approximately 100 mealworms each, while other species were provided wild bird seed. The room lights were left on, but cages were covered with a light-colored sheet. In this manner, birds could not see beyond their cage, but could still locate their perch, food and water.

### Trials

**Figure 1:**
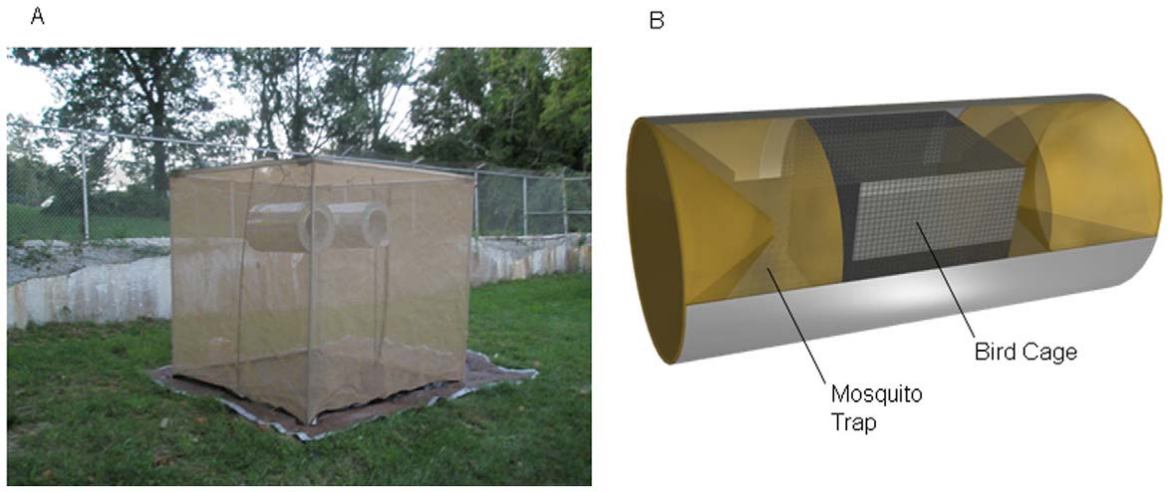
Trial Enclosure (A) and Bird-Baited Trap Design (B).

The host selection trials were conducted with three different species of birds, American robin, European starling, and house sparrow outdoors in two 3×3 m mesh enclosures (Bioquip Products, Inc; Rancho Dominguez, CA 90220) that were erected in an open lawn area away from trees or other obstructions at the Yale Farm in Bethany, CT (Figure 1). The enclosures were mosquito-proofed on all sides by attaching a tarp floor and using durable weather-resistant tape to seal all gaps. A small hole was cut into the side of each enclosure for inserting mosquitoes, and was then plugged to prevent mosquitoes from escaping.

Within each enclosure, two “lard-can” traps [17], [38] baited with a single bird were hung side-by-side separated by one meter. The trap was designed so that mosquitoes could enter from either end but were unable to exit, and contained a mesh screen separating the bird from mosquitoes entering the trap.

The trials were conducted for a 2 hr interval after sunset coincident with peak host-seeking activity reported for *Cx pipiens* [39]. For each trial, one robin and one individual of an alternate species (or a second robin as a control for trap selection bias) were placed within the two lard-can traps. To minimize potential bias due to trap placements, bird placement within the two traps (either left or right trap) was randomized. A trial started as soon as 100–200 (exact number counted for each trial) F1 female *C. pipiens* mosquitoes were inserted into the enclosure. The start and end times were recorded along with the date. Temperature, humidity, and wind speed measurements were taken midway through the trial using a hand-held weather meter (Kestrel® 4000). Mosquitoes remaining in the enclosure after the trial were aspirated using a back-pack aspirator, after which the traps were dismantled, the birds returned to their holding cages and mosquitoes in the collection chambers were frozen and counted. Four American robin-European starling and eight robin-house sparrow trials were conducted. We also ran eight robin-robin pairings, which served as a control.

### Data Analysis

We measured the relative attractiveness of robins over the alternate bird species as the probability of a mosquito entering a robin-baited trap (P_(robin)_) when paired with a trap baited with an alternate bird species.

A logistic regression model was used to examine the effect of covariates on the probability of a mosquito entering a robin-baited trap (Stata/SE 8.0, Stata Co., College Station, TX). The covariates were the alternate individual's species weight, the robin's weight, the weight difference between the robin and the alternate species, the age and sex of birds, the date of the trial, starting-time, temperature, humidity, wind-speed and the age of the mosquitoes.

*C. pipiens*' propensity to engage in host-seeking behavior (‘activation’) was measured as the proportion of mosquitoes that entered either baited trap during each trial. Differences in activation among pairings where assessed using a Chi-square test.

## Results

Host-seeking *C. pipiens* were significantly more attracted to robins than to either sparrows or starlings (Table 1), but no difference was detected when trials were conducted with two robins (Additional information for individual trials are provided in Supplementary Tables S1–S3). *C. pipiens* were three times more likely to enter robin-baited traps when paired with starling-baited traps (OR = 3.06; CI [1.42–6.46]) and almost twice more likely when paired with sparrow-baited traps (OR = 1.80; CI = [1.22–2.90]).

**Table 1:** Results of *C. pipiens* host-choice trials.

Pairing	*n* trials	total *C.* (range/trial)	% activation	P(robin)*	OR (95% CI)**	p value
robin - sparrow	8	1528 (160–208)	12.68±0.83	0.66±0.04	1.80 (1.22–2.90)	0.004
robin - starling	4	710 (145–200)	6.73±1.80	0.76±0.07	3.06 (1.42–6.46)	0.003
robin - robin	8	1400 (100–200)	11.12±2.60	0.47±0.06	0.08 (0.59–1.08)	>0.15

*shows the probability a mosquito enters the robin-baited trap, or the left trap for robin-robin pairings.

**adjusted odds ratios with corresponding 95% confidence intervals.

Multiple logistic regression analyses indicated that the robin's weight, alternate species' weight, the weight difference between the robin and the alternate species, the age and sex of the alternate species, the date of the trial, starting-time, temperature, humidity, wind-speed and the age of the mosquitoes had no effect on the probability of a choosing a robin over an alternate bird.

Activation was 11.1±2.6% for robin-robin pairings (n = 8), 12.6±0.8% for robin-house sparrow pairings (n = 8), 6.7±1.8% for robin-starling pairings, and 7.3±1.3% for two sparrow-sparrow trials not included in regression analysis due to low sample size. The overall percentage of total introduced mosquitoes entering a bird-baited trap (mean activation) was 10.5% and was not significantly different among the host-choice pairings (Chi-square = 5.51, 3df, p = 0.14), indicating that *C. pipiens* activation did not influence preferential host-selection in our study. A larger experiment with more replicates would be helpful to confirm this finding.

## Discussion

Our results indicate that *C. pipiens* display preference for robins when offered a choice between a robin and one of two locally common, sympatric bird species. The degree of preference varied depending on the alternate species, with robins selected approximately three times over European starlings and two times over house sparrows. This preference was not affected by potential confounders such as age of the mosquito cohort, birds' weight, age and sex and differences in body weight between the trial pair and the environmental conditions (temperature, relative humidity and wind speed) in which trials were held. Furthermore no location bias in trap selection was indicated by the equal probability of mosquitoes entering either trap when both were baited with robins. The only covariate which significantly influenced host choice was the species with which the robin was paired. We acknowledge that the current investigation evaluated the attractiveness of three avian species only, and that further experiments with larger numbers will need to be conducted to determine whether a preference for robins is maintained when paired with other common Passeriform species upon which *C. pipiens* is known to feed.

The preference of *C. pipiens* for robins over both European starlings and house sparrows is consistent with studies reporting *C. pipiens*' feeding patterns in the field. Apperson et al [29] reported that although all three birds were among the most abundant species in New Jersey and Tennessee, robins were a common blood source for *C. pipiens*, while house sparrows were only rarely fed upon and no blood meals were identified from European starlings. Feeding indices calculated from blood meal analyses coupled with estimates of the relative abundance of avian species within the local community [30], [35] also suggest that *C. pipiens* preferentially feed on robins. In a study in Maryland, Kilpatrick et al [30] concluded that robins were fed upon 16.4±4.4 (range 6.4–30.6) times more often than would be expected based upon their abundance. In a Chicago, Illinois, study, Hamer et al [35] also found that robins were fed upon more often than their abundance would predict (index 2.26±0.39), while both European starlings and house sparrows were less often fed upon (indices 0.39±0.17 and 0.32±0.05, respectively). High spatial and temporal variability has been reported in field-derived feeding indices, and this may be expected because interactions with complex biotic and abiotic factors in nature modulate the mosquitoes' innate host preferences.

To measure ‘innate’ host-preference by *C. pipiens*, our experimental design used two host-baited traps within a large enclosure to minimize the effect of confounding factors which are often associated with olfactometer and field-based trials. Experiments using laboratory olfactometers or wind-tunnel designs have demonstrated mosquito host-preferences (*Aedes aegypti* L. [40], *Anopheles quadriannulatus* Theobald [41], [42], and reviewed in [43]), but these designs do not represent host-seeking conditions in nature because they often use artificial airstreams, colony-reared mosquitoes, and/or partial host stimuli. In contrast, studies conducted in the field using host-baited traps (*Anopheles gambiae* s.s. Giles [12], *An. gambiae* s.l. *and An. pharoensis* Theobald, [8], *Culex quinquefasciatus* Say[15], *Culex nigripalpus* Theobald [16]) represent natural conditions but cannot control for mosquito densities or account for the presence of other competing hosts.

Our design further provided a measure of host preference, as opposed to measuring feeding success, which may be affected by birds' defensive behaviors which may reduce mosquito feeding success [13], [18], [44]–[46]. It has been documented that starlings will engage in defensive movements or will eat mosquitoes to avoid being bitten [47], and that such behaviors cause biting mosquitoes to divert to more permissive hosts [44]. In our trials, mosquitoes that entered the trap were separated from the bird by a mesh screen.

The 10.5% activation level for *C. pipiens* observed in our study was within the lower ranges reported in olfactometer studies with *An. gambiae* s.s. (*9 to 19%*) [12], and *An. quadriannulatus* (10 to 28%) [45]. Lower activation was most likely due to the absence of artificial airstreams used to induce mosquitoes to engage in appetitive flight.

Innate host preference by *C. pipiens* for particular host species has epidemiological relevance in that preferential feeding by *C. pipiens* on WNV-competent hosts, such as robins, may influence transmission dynamics if contact rates are shifted away from other abundant, but less competent hosts. From the experiments conducted herein, we can derive a potential preference index for *C. pipiens* that can be included in epidemiological models describing WNV enzootic transmission to determine its influence on the dynamics. Current models of WNV enzootic transmission include parameters describing the host community; however, they do not adequately incorporate vector feeding preferences [48]–[53]. Another parameter measured in this experiment that can be used to inform epidemiological models is the activation level, which provides an estimate of the field-deployed mosquito traps' recruitment rate. Finally, estimating innate host preference offers additional benefits to epidemiological studies of vector-host interactions. By providing a fixed estimate of the innate feeding preference, this study allows evaluation of the relative influence of other biotic and abiotic factors. Such comparisons may provide insights into how the environment acts to modulate the innate host preference of vectors.

## Supporting Information

Table S1 Results for individual robin and house sparrow trials.
(0.04 MB DOC)

Table S2 Results of robin and starling trials.
(0.03 MB DOC)

Table S3 Results of the robin and robin control trials.
(0.04 MB DOC)
