# Supplementary material for: Avian Host-Selection by Culex pipiens in Experimental Trials
Source: PLoS One. 2009 Nov 17;4(11):e7861. doi: 10.1371/journal.pone.0007861 (PMC2775674; doi:10.1371/journal.pone.0007861)
Supplement: Table S1 — Results for individual robin and house sparrow trials. (0.04 MB DOC) [file pone.0007861.s001.doc]

| Date | No. *Cx. pipiens* in sparrow-baited trap | No. *Cx. pipiens* in robin-  baited trap | Total *Cx. pipiens* | Age (days) | Time Start | Time End | Probability choosing sparrow-baited trap |
| --- | --- | --- | --- | --- | --- | --- | --- |
| 8/25/2008 | 2 | 28 | 208 | 7 | 19:45 | 21:45 | 0.07 ± 0.05 |
| 9/4/2008 | 14 | 11 | 200 | 10 | 19:45 | 21:45 | 0.56 ± 0.15 |
| 9/4/2008 | 16 | 13 | 200 | 10 | 19:45 | 21:45 | 0.55 ± 0.14 |
| 9/8/2008 | 5 | 14 | 200 | 7 | 20:00 | 22:00 | 0.26 ± 0.12 |
| 9/12/2008 | 6 | 24 | 200 | 8 | 19:30 | 21:30 | 0.20 ± 0.08 |
| 9/15/2008 | 9 | 17 | 200 | 11 | 19:30 | 21:30 | 0.35 ± 0.12 |
| 9/29/2008 | 3 | 11 | 160 | 11 | 19:30 | 21:30 | 0.21 ± 0.12 |
| 9/28/2008 | 11 | 11 | 160 | 11 | 19:30 | 21:30 | 0.50 ± 0.15 |
| Total | 66 | 129 | 1528 |  |  |  | 0.34 ± 0.04 |

**Table S1.** Results of robin and house sparrow trials.
