# Supplementary material for: Avian Host-Selection by Culex pipiens in Experimental Trials
Source: PLoS One. 2009 Nov 17;4(11):e7861. doi: 10.1371/journal.pone.0007861 (PMC2775674; doi:10.1371/journal.pone.0007861)
Supplement: Table S2 — Results of robin and starling trials. (0.03 MB DOC) [file pone.0007861.s002.doc]

| Date | No. *Cx. pipiens* in starling-baited trap | No. *Cx. pipiens* in robin-baited trap | Total *Cx. pipiens* | Age (days) | Time Start | Time End | Probability choosing starling-baited trap |
| --- | --- | --- | --- | --- | --- | --- | --- |
| 8/21/2008 | 1 | 16 | 200 | 5 | 19:30 | 21:30 | 0.06 ± 0.06 |
| 8/27/2008 | 4 | 11 | 145 | 7 | 19:20 | 21:20 | 0.27 ± 0.13 |
| 8/27/2008 | 5 | 5 | 165 | 7 | 19:20 | 21:20 | 0.50 ± 0.22 |
| 9/10/2008 | 1 | 3 | 200 | 8 | 19:30 | 21:30 | 0.25 ± 0.25 |
| Total | 11 | 35 | 710 |  |  |  | 0.24 ± 0.07 |

**Table 2.** Results of robin and starling trials.
