# Supplementary material for: Avian Host-Selection by Culex pipiens in Experimental Trials
Source: PLoS One. 2009 Nov 17;4(11):e7861. doi: 10.1371/journal.pone.0007861 (PMC2775674; doi:10.1371/journal.pone.0007861)
Supplement: Table S3 — Results of the robin and robin control trials. (0.04 MB DOC) [file pone.0007861.s003.doc]

| Date | No. *Cx. pipiens* in Left (L) Trap | No. *Cx. pipiens* in Right (R) trap | Total *Cx. pipiens* | Age (days) | Time Start | Time End | Probability choosing (L) |
| --- | --- | --- | --- | --- | --- | --- | --- |
| 8/15/2008 | 8 | 7 | 100 | 5 | 20:00 | 22:00 | 0.53 ± 0.19 |
| 8/17/2008 | 5 | 6 | 100 | 5 | 19:45 | 21:45 | 0.45 ± 0.20 |
| 8/19/2008 | 6 | 2 | 200 | 4 | 19:30 | 21:30 | 0.75 ± 0.31 |
| 8/22/2008 | 6 | 3 | 200 | 6 | 20:00 | 22:00 | 0.67 ± 0.27 |
| 8/28/2008 | 26 | 20 | 200 | 8 | 20:10 | 22:10 | 0.57 ± 0.11 |
| 9/3/2008 | 5 | 5 | 200 | 10 | 19:45 | 21:45 | 0.50 ± 0.22 |
| 9/15/2008 | 18 | 21 | 200 | 11 | 19:30 | 21:30 | 0.46 ± 0.11 |
| 9/24/2008 | 7 | 7 | 200 | 10 | 19:20 | 21:20 | 0.50 ± 0.19 |
| Total | 81 | 71 | 1400 |  |  |  | 0.53 ± 0.06 |

**Table 3.** Results of the robin and robin control trials.
